# Associations of Neurocognition and Social Cognition With Brain Structure and Function in Early-Onset Schizophrenia

**DOI:** 10.3389/fpsyt.2022.798105

**Published:** 2022-02-10

**Authors:** Pengfei Guo, Shuwen Hu, Xiaolu Jiang, Hongyu Zheng, Daming Mo, Xiaomei Cao, Jiajia Zhu, Hui Zhong

**Affiliations:** ^1^Department of Child and Adolescent Mental Disorder, Affiliated Psychological Hospital of Anhui Medical University, Hefei, China; ^2^Department of Child and Adolescent Mental Disorder, Anhui Mental Health Center, Hefei, China; ^3^Department of Radiology, First Affiliated Hospital of Anhui Medical University, Hefei, China

**Keywords:** early-onset schizophrenia, neurocognition, social cognition, gray matter volume, amplitude of low-frequency fluctuation

## Abstract

**Background:**

Cognitive impairment is a core feature of schizophrenia that is more serious in patients with early-onset schizophrenia (EOS). However, the neuroimaging basis of cognitive functions, including neurocognition and social cognition, remains unclear in patients with EOS.

**Methods:**

Forty-three patients with EOS underwent structural and resting state functional magnetic resonance imaging scans. Brain structure and function were evaluated through the analysis of brain gray matter volume (GMV) and amplitude of low-frequency fluctuations (ALFF). They underwent comprehensive assessments for neurocognition (verbal memory, verbal expression, attention, and executive function) and social cognition (theory of mind and attributional bias). Correlation analyses were conducted to detect the potential link between cognitive function indices and brain imaging parameters.

**Results:**

First, neurocognition was linked to brain structure characterized by higher immediate recall scores associated with increased GMV in the left temporal pole, higher verbal fluency scores associated with increased GMV in the left temporal pole: middle temporal gyrus, and higher Stroop-word scores associated with increased GMV in the right middle frontal gyrus. Second, social cognition was related to brain function characterized by lower sense of reality scores associated with increased ALFF in the left precentral gyrus, higher scores of accidental hostility bias associated with increased ALFF in the right middle temporal gyrus, and higher scores of accidental aggression bias associated with increased ALFF in the left precentral gyrus.

**Conclusion:**

These findings may add to the existing knowledge about the cognitive function-brain relationship. They may have clinical significance for studying the mechanism of neurocognitive and social cognitive impairment in patients with EOS and providing potential neural targets for their treatment and intervention.

## Introduction

Schizophrenia is a chronic and declining psychiatric disorder that affects nearly 1% of the world's population, and commonly occurs in late adolescence or early adulthood (1). Approximately 11% of patients had onset of schizophrenia before the age of 18 (2), which is defined as early-onset schizophrenia (EOS) (3). Compared with adult-onset schizophrenia, adolescents with EOS have significantly worse symptoms and social outcomes characterized by a chronic illness course, insidious onset, and long treatment delays (4, 5). Cognitive dysfunction is a core feature of schizophrenia and can predict functional outcomes (6, 7). Furthermore, after standard treatment with antipsychotic drugs, the cognitive function of patients with schizophrenia cannot be completely improved and may require adjuvant treatments (8, 9). The refractoriness of antipsychotics may be closely related to the early onset of psychotic symptoms. Data from a prospective trial have shown that EOS is more likely to be refractory than adult-onset schizophrenia and have more severe cognitive impairment (10). The results of a longitudinal study have indicated a neurodevelopmental pathway of EOS with subnormal cognitive development specific to adolescence (11). Therefore, a full understanding of the neurobiological basis of cognitive impairment in patients with EOS may have important clinical significance.

In the past, many efforts have been made to explore the mechanisms of cognitive impairment in schizophrenia. It has been reported that cognitive dysfunction in schizophrenia can be improved by stimulating the glycine regulatory site of the N-methyl-d-aspartate-type glutamate receptor (12, 13). Fachim et al. further pointed out that changes in GRIN2B promoter methylation may lead to glutamatergic dysfunction in psychosis and are related to reduced cognitive performance in patients with first-episode schizophrenia (14). Data from electroencephalogram studies revealed that sleep spindle deficits in patients with schizophrenia can predict lower cognitive performance; this includes patients with EOS (15). A previous study suggested that sleep spindle deficits may be caused by dysfunction of the thalamic cortical network (16). A neuroimaging study using diffusion tensor imaging and tractography methods also found that lower fractional anisotropy of the left inferior fronto-occipital fasciculus and left inferior longitudinal fasciculus can predict worse neurocognitive performance in EOS (17). Although these findings contribute to the understanding of the mechanism of cognitive impairment in EOS, the complex association between them still needs further elucidation.

A safe, non-invasive, and easily reproducible neuroimaging approach has been provided for *in vivo* human brain exploration through advances in magnetic resonance imaging (MRI) techniques (18, 19). Voxel-based morphometry (VBM) can be used to analyze the gray matter volume (GMV) in structural MRI (20). The amplitude of low-frequency fluctuation (ALFF) measures the low-frequency oscillation intensity of blood-oxygen-level-dependent (BOLD) time courses in resting-state functional MRI and reflects local neural activity strength (21). Using these methods, some studies have revealed changes in brain structure and function in patients with EOS (22–24). Nevertheless, there is a paucity of structural and functional MRI studies exploring the neural mechanisms of cognitive impairment in patients with EOS. Only a few related studies have reported that the pathophysiological mechanism of widespread cognitive impairment in EOS may be linked to abnormal changes in the GMV (25). For example, Kadriu et al. found that the development of early cognitive deficits in EOS may occur either concomitantly or closely with changes in the hippocampal volume (26). Shi et al. indicated that structural changes and disturbed resting-state functional connectivity in the core empathy network may correlate with the social cognitive deficits in patients with EOS (27). However, these previous studies have been largely limited to a portion of the cognitive functions of patients; they have paid less attention to the global cognitive functions, including neurocognition and social cognition.

A growing body of research showed that cognitive impairment in schizophrenia involves two dimensions: neurocognition and social cognition. Similar to adult-onset cases, EOS patients had neurocognitive and social cognitive impairments (28). Neurocognition is the basic function of the central nervous system, which includes attention and working memory, verbal memory, executive function, thought disorder, and processing speed. Social cognition refers to the ability to recognize and explain the emotions or intentions of others, and the ability to use these social signals to guide conclusions or behaviors, including theory of mind, attribution bias, emotional processing, social knowledge, and social perception (29–32). These dimensions are different but highly correlated (33). Bell et al. suggested that neurocognition leads to different rehabilitation outcomes by affecting social cognition (30).

In this study, neuropsychological tools were used to evaluate the neurocognitive and social cognitive functions of patients with EOS. These included verbal memory and expression, attention and executive function, theory of mind, and attribution bias. VBM and ALFF analyses were used to measure brain structure and function. The associations between global cognitive functions and brain regions in patients with EOS at the level of brain GMV and local neural activity were explored through correlation analyses.

## Materials and Methods

### Participants

This study consisted of 43 Chinese Han, right-handed patients with EOS within a restricted age range of 13–18 years. All patients were recruited consecutively from the Department of Child and Adolescent Mental Disorder, Affiliated Psychological Hospital of Anhui Medical University, Hefei. According to the International Classification of Diseases criteria, two well-trained clinical psychiatrists confirmed patients' diagnosis of EOS. The study's exclusion criteria were as follows: (1) the presence of other psychiatric disorders such as intellectual disability, bipolar disorder, substance-induced mood disorder, anxiety disorder, substance abuse or dependence; (2) a history of significant neurological or physical diseases; and (3) pregnancy or any contraindications for MRI. After a training to ensure consistent methodology of rating, two investigators completed the questionnaire assessment of patients. This study was approved by the Ethics Committee of the Affiliated Psychological Hospital of Anhui Medical University. After a complete description of the study was provided, all participants' legal guardians gave written informed consent. The demographic data of the participants are listed in Table 1 and behavioral outcomes of the cognitive tests are listed in Supplementary Table 1.

**Table 1:** Demographic characteristics.

**Characteristics**	**Mean ±SD**	**Range**
Sex (female/male)	15/28	–
Age (years)	16.63 ± 1.36	13–18
Education (years)	10.37 ± 1.51	7–12
FD (mm)	0.13 ± 0.07	0.04–0.35
TIV (cm^3^)	1401.54 ± 108.84	1186.71–1629.88
Chlorpromazine equivalent doses	389.21 ± 99.93	115.74–528.17
Course of disease (months)	18.77 ± 14.92	2–51
The age of onset of schizophrenia (years)	15.07 ± 4.56	12–18

*SD, standard deviation; FD, frame-wise displacement; TIV, total intracranial volume*.

### Cognition Assessment

#### Neurocognition Assessment

The auditory verbal learning test (AVLT) was performed to evaluate verbal memory function, which included four indices such as immediate recall, short-term delayed recall, long-term delayed recall, and long-term delayed recognition (34). The test contains two different learning materials, each with 15 common Chinese vocabulary items. Participants would score one point for each correct recall of a word. In the immediate recall test, participants listened to the first set of words five times and recalled the words they heard within 2 min after each listening. Investigators recorded the score each time and calculated the average score as “immediate recall.” Then, participants listened to the second set of words and immediately recalled it within 2 min after listening. After that, participants were asked to recall the first set of words, and investigators recorded the scores as “short-term delayed recall.” Thirty minutes after the fifth listening session, participants needed to recall the first set of words again, and the scores were noted as the “long-term delayed recall.” Finally, participants were asked to listen to a 50-word vocabulary consisting of the first set of words, the second set of words and other interference words, and recognized the words in the first set. The score recognized successfully was used as an index of “long-term delayed recognition.”

The verbal fluency test (VFT) was used to assess verbal expression (35). Each participant had to list as many names as possible of household appliances, animals, and fruits within 1 min, respectively. Participants would score one point for each name. The investigators recorded the total scores as an index of verbal expression performance.

Attention was evaluated using the digit span tasks (36). The tasks consisted of two indices such as digit span forward and digit span backward. Span is defined as the maximum number of digits repeated by the participant. All participants completed a digit span forward task followed by a digit-span backward task. The former begins with a series of two digits orally presented to each participant, continuing to a maximum of 13 digits. Participants were asked to repeat the digits verbally. There were two trials per digit series. All participants began with the first digit series (i.e., two digits). If repeated correctly, the participant continued to the next one; otherwise, the second trial was performed with the same digit series. The task was discontinued when the participant failed in the second trial. The digit span backward task followed the same procedure, except that participants verbally repeated the digits in reverse order.

The Stroop color word test reflects executive function (37). There were three indices including Stroop-dot, Stroop-word and Stroop-color word in the test. The test materials consisted of three types of cards: A (dot), B (word), and C (color word). Card A had red, green, yellow, and blue dots arranged in a certain order in a 4 × 4 manner, and card B had four Chinese words (namely “Yin,” “Zou,” “Wen,” “Sheng”) with four colors of red, green, yellow, and blue arranged in the same order. Card C had four Chinese words (namely “red,” “green,” “yellow,” “blue”) with four colors of red, green, yellow, and blue arranged in the order of card A. The participants were asked to quickly read the color of the dots or Chinese words on the cards. The time taken to read each card was used as an index of executive function performance.

#### Social Cognition Assessment

The Theory of Mind Picture-Sequencing Task was performed to assess the ability of mentalization (38) through six stories. Each story consisted of four pictures, accompanied by 2~6 questions. Participants composed a story with four pictures in a certain logical order and then answered questions from the investigators. Participants would get two points, respectively for the first and last pictures sequenced correctly of each story and get one point, respectively for the two pictures in the middle of each story. At the same time, one point would be awarded for each correct answer. These questions assessed the participants' ability to understand the mental state of the characters in the story, including primary beliefs, primary false beliefs, secondary beliefs, secondary false beliefs, tertiary false beliefs, sense of reality, understanding reciprocity, understanding deception, and detecting deception. Adding the total score, there were ten indices extracted from the test. The maximum score was 59. The scores were used as indices of mentalizing ability.

The Ambiguous Intentions Hostility Questionnaire (AIHQ) was used to evaluate attribution bias (39). The Chinese version of AIHQ is composed of three types of hypothetical scenarios describing the characters' behaviors: intentional (five vignettes), ambiguous (five vignettes), or accidental (five vignettes). After reading each vignette, participants were asked to imagine the scenario happening to them (e.g., “You walk past a bunch of teenagers at a mall and you hear them start to laugh”), and to write down why the other person (or persons) treated you this way. Two independent investigators subsequently rated this written response for the purpose of computing a “hostility index.” Then, the participants rated, on Likert scales, whether the other person (or persons) performed the action on purpose (1 “definitely no” to 6 “definitely yes”), how angry it would make them feel (1 “not at all angry” to 5 “very angry”), and how much they would blame the other person (or persons) (1 “not at all” to 5 “very much”). Investigators calculated the average score of these three items as the “blame index.” Finally, the participants were asked to write down how they would respond to the situation, which was later rated by two independent investigators to compute an “aggression index.” For the hostility and aggression indices, two investigators independently evaluated each participant's reaction on 5-point Likert scales based on examples of high and low scores. The scales for the hostility and aggression indices were from 1 (“not at all hostile”) to 5 (“very hostile”), and 1 (“not at all aggressive”) to 5 (“very aggressive”), respectively. The hostility bias, blame bias, and aggression bias scores for each scenario type were averaged after being added up, respectively. Furthermore, the total scores for each bias in the three types of scenarios were added up as the total scores of hostility bias, total scores of blame bias, and total scores of aggression bias. On the whole, there were 12 indices extracted from the test. Higher scores indicated a more negative attribution bias.

### MRI Data Acquisition

MRI scans were obtained using a 3.0-Tesla MR system (Discovery MR750w, General Electric, Milwaukee, WI, USA) with a 24-channel head coil. Earplugs were used to reduce scanner noise, and tight but comfortable foam padding was used to minimize head motion. Before the scanning, all participants were instructed to keep their eyes closed, think of nothing in particular, move as little as possible, and relax but not fall asleep during the scans. High-resolution 3D T1-weighted structural images were acquired by employing a brain volume (BRAVO) sequence with the following parameters: repetition time (TR) = 8.5 ms; echo time (TE) = 3.2 ms; inversion time = 450 ms; flip angle (FA) = 12°; field of view (FOV) = 256 mm × 256 mm; matrix size = 256 × 256; slice thickness = 1 mm, no gap; voxel size = 1 mm × 1 mm × 1 mm; 188 sagittal slices. Resting-state BOLD fMRI data were acquired using a gradient-echo single-shot echo planar imaging (GRE-SS-EPI) sequence with the following parameters: TR = 2,000 ms; TE = 30 ms; FA= 90°; FOV = 220 mm × 220 mm; matrix size = 64 ×64; slice thickness = 3 mm, slice gap = 1 mm; 35 interleaved axial slices; voxel size = 3 mm × 3 mm × 3 mm; 185 volumes. After the scanning, all images were visually inspected to ensure that only images without visible artifacts, lesions, and regional deformations were included in subsequent analyses.

### Gray Matter Volume Analysis

The 3D T1-weighted structural images were processed using the VBM8 toolbox (http://dbm.neuro.uni-jena.de/vbm.html) implemented in the Statistical Parametric Mapping software (SPM8, http://www.fil.ion. ucl.ac.uk/spm). First, all the structural images were segmented into gray matter, white matter, and cerebrospinal fluid density maps using the standard segmentation model. After an initial affine registration of the gray matter density map into the Montreal Neurological Institute (MNI) space, the gray matter density images were non-linearly warped using the diffeomorphic anatomical registration through the exponentiated lie algebra (DARTEL) technique (40). They were then resampled to a voxel size of 1.5 mm × 1.5 mm × 1.5 mm. The GMV map was obtained by multiplying the gray matter density map by the non-linear determinants derived from the spatial normalization step. Finally, the resultant GMV images were smoothed with a Gaussian kernel of 8 mm × 8 mm × 8 mm full-width at half maximum.

### ALFF Analysis

Resting-state BOLD data were preprocessed using SPM12 (http://www.fil.ion.ucl.ac.uk/spm) and Data Processing & Analysis for Brain Imaging (DPABI, http://rfmri.org/ dpabi) (41). The first ten volumes for each participant were discarded to allow the signal to reach equilibrium and the participants to adapt to the scanning noise. The remaining volumes were corrected for the acquisition time delay between slices. Then, realignment was performed to correct the motion between the time points. Head motion parameters were computed by estimating the translation in each direction and the angular rotation on each axis for each volume. All participants' BOLD data were within the defined motion thresholds (i.e., translational or rotational motion parameters <2 mm or 2°, respectively). The frame-wise displacement (FD), which indexes the volume-to-volume changes in the head position, was also calculated. Several nuisance covariates (the linear drift, estimated motion parameters based on the Friston-24 model, spike volumes with FD > 0.5, white matter signal, and cerebrospinal fluid signal) were regressed out from the data. In the normalization step, individual structural images were first co-registered with the mean functional image; thereafter, the transformed structural images were segmented and normalized to the MNI space using the diffeomorphic anatomical registration through the exponentiated lie algebra (DARTEL) technique (40). Finally, each functional volume was spatially normalized to the MNI space using the deformation parameters estimated during the above step and resampled into a 3 mm cubic voxel. After spatial normalization, all data sets were smoothed with a 6 mm full-width at half maximum Gaussian kernel.

ALFF analysis was conducted using DPABI software (http://rfmri.org/ dpabi). After preprocessing, each voxel's BOLD time course was filtered (bandpass, 0.01–0.1 Hz) to remove the effects of very-low-frequency drift and high frequency noise, e.g., respiratory and heart rhythms. The fast Fourier transform with default parameters from DPABI was used to transform the filtered BOLD time course of each voxel into frequency domain and the power spectrum was then obtained. Because the power of a given frequency is proportional to the square of the amplitude of this frequency component of the original BOLD time course in the time domain, the square root was calculated at each frequency of the power spectrum at each voxel. The averaged squared root was obtained across 0.01–0.1 Hz, which was defined as the ALFF value. Finally, the ALFF value of each voxel was divided by the global mean ALFF value for standardization.

### Statistical Analysis

The statistical descriptive analyses of demographic and behavioral data were conducted using the SPSS software (version 23.0; SPSS, Chicago, IL, USA). This study examined the relationship between cognitive function indices and brain imaging parameters in a voxel-wise manner within the whole gray matter in EOS patients. The general linear model in Statistical Parametric Mapping software (SPM12, http://www.fil.ion.ucl.ac.uk/spm) with a multiple regression design was used to identify any voxels in the GMV and ALFF maps that showed a significant association with the behavioral outcomes while controlling for age, sex, and years of education. Total intracranial volume (TIV) and FD were additional covariates for the GMV and ALFF analyses, respectively. The resulting maps were thresholded at an uncorrected voxelwise level of *p* < 0.001, and then considered significant at *p* < 0.05 cluster-level family wise error (FWE)-corrected for multiple comparisons. For each subject, the GMV and ALFF values of each cluster with a significant correlation with behavioral outcomes were extracted by the RESTplus software (http://www.restfmri.net), and then used for region of interest (ROI)-based analyses that refers to the partial correlation analyses performed in SPSS while controlling the above covariates (Supplementary Tables 2, 3). To ensure the robustness of the findings, this study repeated the ROI-based partial correlation analyses after removing the outliers of outcomes (GMA and ALFF values, and behavioral outcomes) greater than mean + 3 × standard deviation (SD) or smaller than mean – 3 × SD. No corrections for multiple testing were conducted, as the objective for this study was to generate some hypotheses for further testing and confirmation in a larger sample.

### Sensitivity Analysis

First, a previous study revealed the effect of antipsychotic drugs on brain structure and function (42). To test the possible effect of antipsychotic drugs on our results, chlorpromazine equivalent doses for antipsychotics were included as an additional nuisance covariate with repetition in the ROI-based partial correlation analyses (43). Second, the age of onset of schizophrenia might affect GMV and ALFF, so the ROI-based partial correlation analyses were repeated while adding this variable as an additional nuisance covariate.

## Results

### Correlations Between Neurocognition and GMV

**Figure 1:**
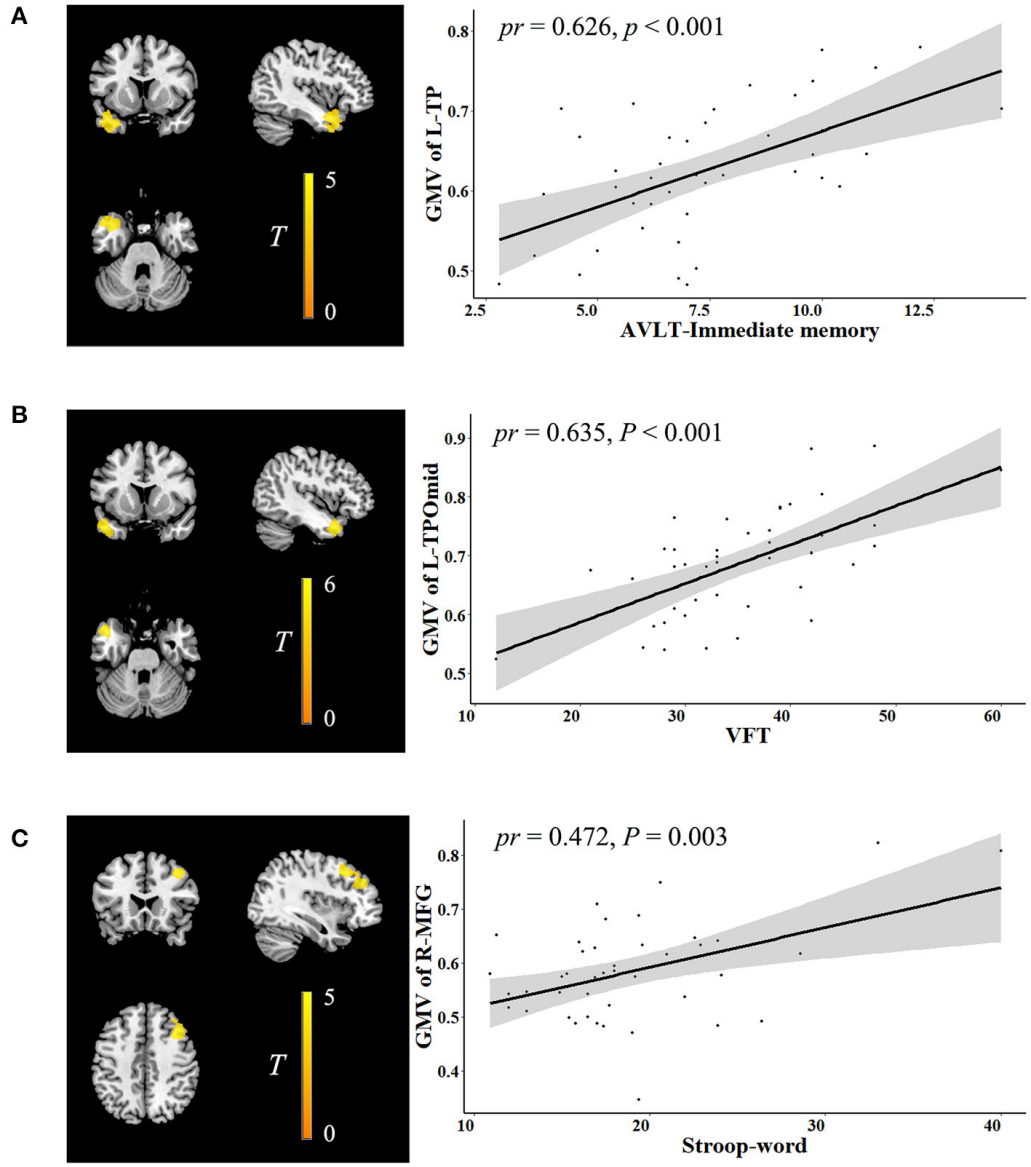
Correlation between neurocognition and GMV. The left figure in **(A–C)** is voxel-based and the right is ROI-based. **(A)** Correlation between GMV in the left TP and AVLT-immediate recall. **(B)** Correlation between GMV in the left TPOmid and VFT. **(C)** Correlation between GMV in the right MFG and stroop-word. GMV, gray matter volume; ROI, region of interest; TP, temporal pole; AVLT, auditory verbal learning test; TPOmid, temporal pole: middle temporal gyrus; VFT, verbal fluency test; MFG, middle frontal gyrus; *pr*, partial correlation coefficient; L, left; R, right.

In the voxel-wise whole gray matter analysis, the significant correlations between neurocognition and GMV are shown in Figure 1 (cluster-level *P* < 0.05, FWE corrected). After adjustment for age, sex, years of education, TIV, and outliers, significant positive correlations were found between the AVLT-immediate recall and GMV in the left temporal pole (TP) (cluster size: 1,602 voxels, peak MNI coordinate x/y/z: −39/4.5/-30, peak *T*: 4.872, partial correlation coefficient [*pr*]: 0.626, *P* < 0.001); positive correlations between the VFT and GMV in the left TP: middle temporal gyrus (cluster size: 762 voxels, peak MNI coordinate x/y/z: −46.5/12/-30, peak *T*: 5.319, *pr*: 0.635, *P* < 0.001); and positive correlations between the Stroop-word and GMV in the right middle frontal gyrus (MFG) (cluster size: 1,289 voxels, peak MNI coordinate x/y/z: 40.5/22.5/42, peak *T*: 4.951, *pr*: 0.472, *P* = 0.003). No significant correlations were observed between neurocognition and ALFF.

### Correlations Between Social Cognition and ALFF

**Figure 2:**
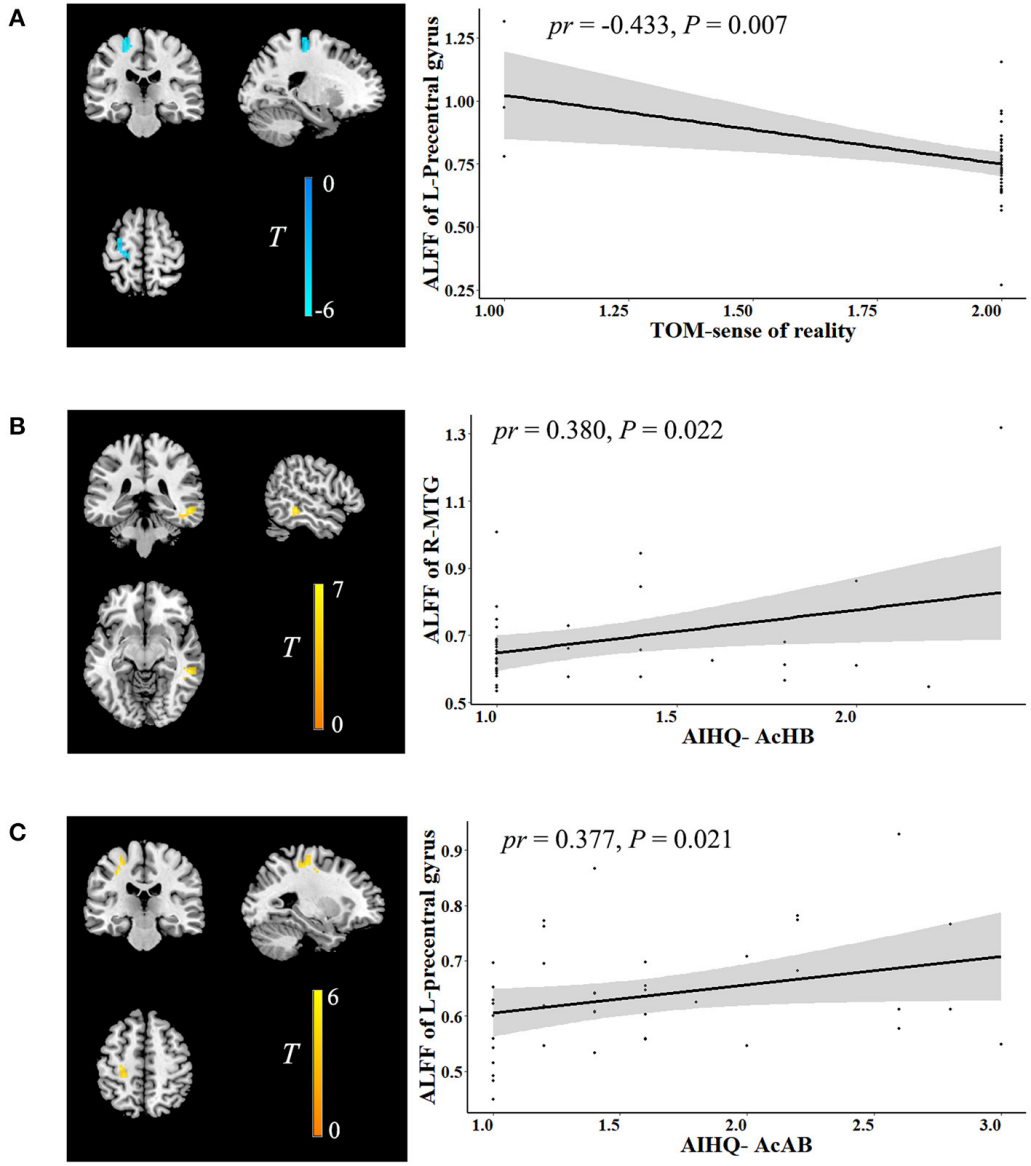
Correlation between social cognition and ALFF. The left figure in **(A–C)** is voxel-based and the right is ROI-based. **(A)** Correlation between ALFF in the left precentral gyrus and TOM-sense of reality. **(B)** Correlation between ALFF in the right MTG and AIHQ-AcHB. **(C)** Correlation between ALFF in the left precentral gyrus and AIHQ-AcAB. ALFF, amplitude of low-frequency fluctuation; ROI, region of interest; TOM, theory of mind; AIHQ, ambiguous intentions hostility questionnaire; AcHB, accidental hostility bias; MTG, middle temporal gyrus; AcAB, accidental aggression bias; *pr*, partial correlation coefficient; L, left; R, right.

In the ALFF analysis, the significant correlations between social cognition and ALFF are shown in Figure 2 (cluster-level *P* < 0.05, FWE corrected). After accounting for age, sex, years of education, FD, and outliers, the Theory of Mind-sense of reality showed significant negative correlations with ALFF in the left precentral gyrus (cluster size: 67 voxels, peak MNI coordinate x/y/z: −21/-24/66, peak *T*: −5.973, *pr*: −0.433, *P* = 0.007). The accidental hostility bias exhibited significant positive correlations with ALFF in the right middle temporal gyrus (MTG) (cluster size: 51 voxels, peak MNI coordinate x/y/z: 54/-36/-9, peak *T*: 6.187, *pr*: 0.380, *P* = 0.022). There were significant positive correlations between the accidental aggression bias and ALFF in the left precentral gyrus (cluster size: 51 voxels, peak MNI coordinate x/y/z: −30/-24/45, peak *T*: 5.854, *pr*: 0.377, *P* = 0.021). No significant correlations were observed between social cognition and GMV.

### Sensitivity Analysis

First, after considering the influence of antipsychotics, the correlations between cognitive function indices and brain imaging parameters had slight changes in p values, but the main brain regions in the results had survived (Supplementary Tables 4, 5). Second, the correlations between cognitive function indices and brain imaging parameters remained unchanged after additionally adjusting for the age of onset of schizophrenia (Supplementary Tables 6, 7). These suggest that antipsychotics and the age of onset of schizophrenia did not influence the main results of this study.

## Discussion

Using structural and resting-state functional MRI, we explored the relationships between neurocognitive function, social cognitive function, and brain imaging parameters in patients with EOS. There were two main findings in this study. The first was the relationship between neurocognitive function and brain structure. Higher language functions of verbal memory and expression were associated with increased GMV in the TP. Meanwhile, higher executive function was associated with increased GMV in the MFG. The second was the relationship between social cognitive function and brain function. This was characterized by lower theory of mind ability associated with increased ALFF in the precentral gyrus and more negative attribution bias with increased ALFF in the precentral gyrus, and MTG.

Previous studies have used VBM and ALFF methods to investigate changes in GMV and local neural activity in patients with schizophrenia (44–48). The TP, MFG, and MTG are the common brain areas consistently reported in previous studies and the current study. For instance, compared with healthy participants, some studies found that patients with schizophrenia demonstrate decreased GMV in the MFG and paralimbic system (including the orbitofrontal cortex, insular cortex, and TP) (44, 45). Li et al. found differences in the variability of dynamic ALFF between EOS patients and healthy controls in the bilateral precuneus, right superior marginal gyrus, right postcentral gyrus, and right MTG (48). However, the investigators focused solely on distinguishing this disease and paid no attention to changes in the global cognitive function of patients. We speculate that not only do EOS patients have altered GMV or local nerve activity in the common regions, but these alterations may also affect neurocognitive and social cognitive functions. There are some inconsistent findings between the current and previous studies. The differences across studies may be linked to patient characteristics (subtype of schizophrenia, age of onset, and course of disease), brain imaging methods (GMV, ALFF, and dynamic ALFF), and sample sizes.

The TP is the most rostral part of the temporal lobe, and its complex structure has been associated with a variety of advanced cognitive functions, including autobiographic memory (49–51), semantic memory (52, 53), generation of false memories (54), face processing (55) and processing of emotional understanding (56, 57). The “semantic hub” theory is one important theory about TP function, which considers the TP as a domain-general hub integrating semantic information from different modalities into a coherent representation (58, 59). Pehrs et al. showed that the TP plays a role in the integration of various semantic information and top-down modulation in the ventral visual stream (57). In patients with Alzheimer's disease, impaired semantic performance was associated with decreased GMV in the TP (60). Similarly, impaired semantic memory was also found in patients with epileptogenic lesions at the left TP (61). Consistent with these prior findings, a correlation between the TP and language function in EOS patients was found in this study. This suggests that damage to the TP may be a shared underlying neural mechanism leading to language dysfunction in a variety of neuropsychiatric diseases. The MFG is considered to be a component of the central executive network and involves processing of working memory information and the judgment and decision of goal-oriented behaviors (62–64). There is neuropsychological evidence that executive functions of schizophrenia, such as working memory and planning, are impaired (65), as well as neuroimaging evidence that specific deficits in an executive function known as goal maintenance in patients with schizophrenia are associated with reduced MFG activity (66). Collectively, these prior findings, coupled with the results of this study, suggest that MFG dysfunction may serve as the basis of executive function deficits in patients with schizophrenia, including EOS.

One interesting aspect of the results of this study was the negative correlation between social cognitive performance and local neural activity in the precentral gyrus and MTG in patients with EOS. The precentral gyrus is considered to be the core area of the mirror-neuron system and plays a fundamental role in both action understanding and imitation (67), associated with the realization of a variety of social cognitive functions (68, 69). Previous studies found that the aberrant connectivity of the mirror neuron system network may be linked to social dysfunction in patients with schizophrenia, and this aberrant connectivity might even exist in the early stages of psychosis (70, 71). Together with our data, it is reasonable to assume that functional deficits of the precentral gyrus may be a neural characteristic of the impaired social cognition in EOS patients. The MTG plays a vital role in social cognition, semantic processing, auditory processing, action observation and language (72). Increasing number of studies have reported that the MTG is also an important component of the Theory of Mind network. For example, Schurz et al. found significant activation in the MTG during the tasks of social animations, mind in the eyes, and rational actions (73). Another study reported that, compared with false beliefs, social animations showed the strongest activation in the MTG (74). In patients with schizophrenia, a diffusion-weighted MRI study reported an increased trace in the right MTG and a correlation between trace and decreased social cognition (75). Taken together, our results support the idea that MTG dysfunction serves as the substrate underlying impaired social cognition in the EOS.

There are some limitations that should be noted in this study. First, healthy controls were not included in this study. Although our results are similar to those of previous controlled studies with healthy controls, future investigations are warranted to further strengthen our findings more rigorously and accurately by enrolling a sample of subjects with EOS and healthy adolescents. Second, causal relationships cannot be inferred from this cross-sectional design. Longitudinal studies with interventions targeting the improvement of cognitive function in EOS are required to establish the direction of causality. Third, the patients were receiving antipsychotic medication that may have influenced the interpretation of results. Although the main results remained after adjusting for antipsychotic dose equivalents, the medication effect could not be completely eliminated (43). Future studies with medication-naïve first-episode patients with EOS are needed to validate the preliminary findings of this study. Fourth, other indices (e.g., processing speed and emotional processing) would need to be analyzed to further clarify the cognitive function-brain relationship in the EOS. Finally, the results of this study were not corrected for multiple comparisons, because the results might not survive correction, likely due to the small sample size. However, our analyses were important for future hypothesis generation. Future studies are required to further expand the sample size and conduct the correction for multiple comparisons to make the results more reliable.

In conclusion, the present study is the first to explore the association between global cognitive function and the brain in patients with EOS. The observed relationships of neurocognition with GMV and social cognition with ALFF may help to expand the existing knowledge about the cognitive function-brain relationship in EOS. To some extent, this disassociation between anatomy and function also supports that neurocognition is the basic cognitive function that affects the ability of daily living, and social cognition, which is a more advanced cognitive function, can better predict social outcome (29). More broadly, these findings may have clinical significance for studying the neurological mechanism of cognitive impairment in patients with EOS and provide potential neural targets for their treatment and intervention.

## Data Availability Statement

The original contributions presented in the study are included in the article/Supplementary Material, further inquiries can be directed to the corresponding author/s.

## Ethics Statement

The studies involving human participants were reviewed and approved by the Ethics Committee of the Affiliated Psychological Hospital of Anhui Medical University. Written informed consent to participate in this study was provided by the participants' legal guardian/next of kin.

## Author Contributions

PG and SH: methodology, data curation, software, and writing original draft. XJ, HoZ, DM, and XC: data collection, visualization, and investigation. JZ: conceptualization, methodology, software, and formal analysis. HuZ: conceptualization, supervision, and writing—review and editing. All authors contributed to the article and approved the submitted version.

## Funding

This work was supported by the grants of Anhui Provincial Department of Science and Technology (No. 1804h08020251) and National Key Research and Development Program (No. 2018YFC1314300).

## Conflict of Interest

The authors declare that the research was conducted in the absence of any commercial or financial relationships that could be construed as a potential conflict of interest. The Handling Editor YT declared a shared affiliation, though no other collaboration, with one of the authors PG, SH, XJ, HoZ, DM, XC, JZ, and HuZ at the time of the review.

## Publisher's Note

All claims expressed in this article are solely those of the authors and do not necessarily represent those of their affiliated organizations, or those of the publisher, the editors and the reviewers. Any product that may be evaluated in this article, or claim that may be made by its manufacturer, is not guaranteed or endorsed by the publisher.

## Abbreviations

AIHQ: Ambiguous Intentions Hostility Questionnaire
ALFF: amplitude of low-frequency fluctuation
AVLT: auditory verbal learning test
BOLD: blood-oxygen-level-dependent
DARTEL: diffeomorphic anatomical registration through the exponentiated lie algebra
DPABI: Data Processing & Analysis for Brain Imaging
EOS: early onset schizophrenia
FA: flip angle
FD: frame-wise displacement
FOV: field of view
FWE: family wise error
GMV: gray matter volume
MFG: middle frontal gyrus
MNI: Montreal Neurological Institute
MRI: magnetic resonance imaging
MTG: middle temporal gyrus
*Pr*: partial correlation coefficient
ROI: region of interest
rTPJ: right temporoparietal junction
SD: standard deviation
TE: echo time
TIV: total intracranial volume
TP: temporal pole
TR: repetition time
VBM: voxel-based morphometry
VFT: verbal fluency test.

## References

[1] Asarnow JR Tompson MC McGrath EP. Annotation: childhood-onset schizophrenia: clinical and treatment issues. J Child Psychol Psychiatry. (2004) 45:180–94. 10.1111/j.1469-7610.2004.00213.x 14982235

[2] Amminger GP Henry LP Harrigan SM Harris MG Alvarez-Jimenez M Herrman H. Outcome in early-onset schizophrenia revisited: findings from the Early Psychosis Prevention and Intervention Centre long-term follow-up study. Schizophr Res. (2011) 131:112–9. 10.1016/j.schres.2011.06.009 21741219

[3] Werry JS. Child and adolescent (early onset) schizophrenia: a review in light of DSM-III-R. J Autism Dev Disord. (1992) 22:601–24. 10.1007/BF01046330 1483979

[4] Joa I Johannessen JO Langeveld J Friis S Melle I Opjordsmoen S. Baseline profiles of adolescent vs. adult-onset first-episode psychosis in an early detection program. Acta Psychiatr Scand. (2009) 119:494–500. 10.1111/j.1600-0447.2008.01338.x 19207132

[5] Hollis C. Adult outcomes of child- and adolescent-onset schizophrenia: diagnostic stability and predictive validity. Am J Psychiatry. (2000) 157:1652–9. 10.1176/appi.ajp.157.10.1652 11007720

[6] Reichenberg A Harvey PD. Neuropsychological impairments in schizophrenia: integration of performance-based and brain imaging findings. Psychol Bull. (2007) 133:833–58. 10.1037/0033-2909.133.5.833 17723032

[7] Harvey PD Howanitz E Parrella M White L Davidson M Mohs RC. Symptoms, cognitive functioning, and adaptive skills in geriatric patients with lifelong schizophrenia: a comparison across treatment sites. Am J Psychiatry. (1998) 155:1080–6. 10.1176/ajp.155.8.1080 9699697

[8] Keefe RS Bilder RM Davis SM Harvey PD Palmer BW Gold JM. Neurocognitive effects of antipsychotic medications in patients with chronic schizophrenia in the CATIE Trial. Arch Gen Psychiatry. (2007) 64:633–47. 10.1001/archpsyc.64.6.633 17548746

[9] Swartz MS Perkins DO Stroup TS Davis SM Capuano G Rosenheck RA. Effects of antipsychotic medications on psychosocial functioning in patients with chronic schizophrenia: findings from the NIMH CATIE study. Am J Psychiatry. (2007) 164:428–36. 10.1176/ajp.2007.164.3.428 17329467

[10] Iasevoli F Razzino E Altavilla B Avagliano C Barone A Ciccarelli M. Relationships between early age at onset of psychotic symptoms and treatment resistant schizophrenia. Early Interv Psychiatry. (2021). 10.1111/eip.13174. [Epub ahead of print]. 33998142 PMC9291026

[11] Oie MG Sundet K Haug E Zeiner P Klungsoyr O Rund BR. Cognitive performance in early-onset schizophrenia and attention-deficit/hyperactivity disorder: a 25-year follow-up study. Front Psychol. (2020) 11:606365. 10.3389/fpsyg.2020.606365 33519613 PMC7841368

[12] Javitt DC. Glutamate as a therapeutic target in psychiatric disorders. Mol Psychiatry. (2004) 9:984–97, 79. 10.1038/sj.mp.4001551 15278097

[13] Tsai GE Yang P Chang YC Chong MY. D-alanine added to antipsychotics for the treatment of schizophrenia. Biol Psychiatry. (2006) 59:230–4. 10.1016/j.biopsych.2005.06.032 16154544

[14] Fachim HA Loureiro CM Corsi-Zuelli F Shuhama R Louzada-Junior P Menezes PR. GRIN2B promoter methylation deficits in early-onset schizophrenia and its association with cognitive function. Epigenomics. (2019) 11:401–10. 10.2217/epi-2018-0127 30785307

[15] Ferrarelli F Tononi G. Reduced sleep spindle activity point to a TRN-MD thalamus-PFC circuit dysfunction in schizophrenia. Schizophr Res. (2017) 180:36–43. 10.1016/j.schres.2016.05.023 27269670 PMC5423439

[16] Bartho P Slezia A Matyas F Faradzs-Zade L Ulbert I Harris KD. Ongoing network state controls the length of sleep spindles via inhibitory activity. Neuron. (2014) 82:1367–79. 10.1016/j.neuron.2014.04.046 24945776 PMC4064116

[17] Epstein KA Cullen KR Mueller BA Robinson P Lee S Kumra S. White matter abnormalities and cognitive impairment in early-onset schizophrenia-spectrum disorders. J Am Acad Child Adoles Psychiatry. (2014) 53:362–72 e1–2. 10.1016/j.jaac.2013.12.007 24565363 PMC3977613

[18] Lui S Zhou XJ Sweeney JA Gong Q. Psychoradiology: the frontier of neuroimaging in psychiatry. Radiology. (2016) 281:357–72. 10.1148/radiol.2016152149 27755933 PMC5084981

[19] Lerch JP van der Kouwe AJ Raznahan A Paus T Johansen-Berg H Miller KL. Studying neuroanatomy using MRI. Nat Neurosci. (2017) 20:314–26. 10.1038/nn.4501 28230838

[20] Ashburner J Friston KJ. Voxel-based morphometry–the methods. Neuroimage. (2000) 11:805–21. 10.1006/nimg.2000.0582 10860804

[21] Zang YF He Y Zhu CZ Cao QJ Sui MQ Liang M. Altered baseline brain activity in children with ADHD revealed by resting-state functional MRI. Brain Dev. (2007) 29:83–91. 10.1016/j.braindev.2006.07.002 16919409

[22] Tordesillas-Gutierrez D Koutsouleris N Roiz-Santianez R Meisenzahl E Ayesa-Arriola R Marco de Lucas E. Grey matter volume differences in non-affective psychosis and the effects of age of onset on grey matter volumes: a voxelwise study. Schizophrenia research. (2015) 164:74–82. 10.1016/j.schres.2015.01.032 25687531

[23] Tang J Liao Y Zhou B Tan C Liu W Wang D. Decrease in temporal gyrus gray matter volume in first-episode, early onset schizophrenia: an MRI study. PLoS ONE. (2012) 7:e40247. 10.1371/journal.pone.0040247 22802957 PMC3388989

[24] Zheng J Zhang Y Guo X Duan X Zhang J Zhao J. Disrupted amplitude of low-frequency fluctuations in antipsychotic-naive adolescents with early-onset schizophrenia. Psychiatry Res Neuroimaging. (2016) 249:20–6. 10.1016/j.pscychresns.2015.11.006 27000303

[25] Gao ZT Li YL Guo SQ Xia YH. [Brain gray matter volume alterations and cognitive function in first-episode childhood-and adolescence-onset schizophrenia]. Zhonghua Yi Xue Za Zhi. (2019) 99:3581–6. 10.3760/cma.j.issn.0376-2491.2019.45.010 31826575

[26] Kadriu B Gu W Korenis P Levine JM. Do cognitive and neuropsychological functioning deficits coincide with hippocampal alteration during first-psychotic episode? CNS Spectr. (2019) 24:472–8. 10.1017/S1092852918001293 30507369

[27] Shi LJ Zhou HY Wang Y Shen YM Fang YM He YQ. Altered empathy-related resting-state functional connectivity in adolescents with early-onset schizophrenia and autism spectrum disorders. Asian J Psychiatr. (2020) 53:102167. 10.1016/j.ajp.2020.102167 32474345

[28] Harvey PD Isner EC. Cognition, social cognition, and functional capacity in early-onset schizophrenia. Child Adolesc Psychiatr Clin N Am. (2020) 29:171–82. 10.1016/j.chc.2019.08.008 31708046

[29] Silberstein J Harvey PD. Cognition, social cognition, and Self-assessment in schizophrenia: prediction of different elements of everyday functional outcomes. CNS Spectr. (2019) 24:88–93. 10.1017/S1092852918001414 30683165 PMC6414257

[30] Bell M Tsang HW Greig TC Bryson GJ. Neurocognition, social cognition, perceived social discomfort, and vocational outcomes in schizophrenia. Schizophr Bull. (2009) 35:738–47. 10.1093/schbul/sbm169 18245058 PMC2696363

[31] Wexler BE Bell MD. Cognitive remediation and vocational rehabilitation for schizophrenia. Schizophr Bull. (2005) 31:931–41. 10.1093/schbul/sbi038 16079390

[32] Green MF Penn DL Bentall R Carpenter WT Gaebel W Gur RC. Social cognition in schizophrenia: an NIMH workshop on definitions, assessment, and research opportunities. Schizophr Bull. (2008) 34:1211–20. 10.1093/schbul/sbm145 18184635 PMC2632490

[33] Sergi MJ Rassovsky Y Widmark C Reist C Erhart S Braff DL. Social cognition in schizophrenia: relationships with neurocognition and negative symptoms. Schizophr Res. (2007) 90:316–24. 10.1016/j.schres.2006.09.028 17141477

[34] Van der Elst W van Boxtel MP van Breukelen GJ Jolles J. Rey's verbal learning test: normative data for 1855 healthy participants aged 24-81 years and the influence of age, sex, education, and mode of presentation. J Int Neuropsychol Soc. (2005) 11:290–302. 10.1017/S1355617705050344 15892905

[35] Vasquez BP Zakzanis KK. The neuropsychological profile of vascular cognitive impairment not demented: a meta-analysis. J Neuropsychol. (2015) 9:109–36. 10.1111/jnp.12039 24612847

[36] Groth-Marnat G Baker S. Digit Span as a measure of everyday attention: a study of ecological validity. Percept Mot Skills. (2003) 97:1209–18. 10.2466/pms.2003.97.3f.1209 15002866

[37] Houx PJ Jolles J Vreeling FW. Stroop interference: aging effects assessed with the Stroop Color-Word Test. Exp Aging Res. (1993) 19:209–24. 10.1080/03610739308253934 8223823

[38] Van Rheenen TE Rossell SL. Picture sequencing task performance indicates theory of mind deficit in bipolar disorder. J Affect Disord. (2013) 151:1132–4. 10.1016/j.jad.2013.07.009 23916306

[39] Combs DR Penn DL Wicher M Waldheter E. The Ambiguous Intentions Hostility Questionnaire (AIHQ): a new measure for evaluating hostile social-cognitive biases in paranoia. Cogn Neuropsychiatry. (2007) 12:128–43. 10.1080/13546800600787854 17453895

[40] Ashburner J. A fast diffeomorphic image registration algorithm. Neuroimage. (2007) 38:95–113. 10.1016/j.neuroimage.2007.07.007 17761438

[41] Yan CG Wang XD Zuo XN Zang YF DPABI. Data processing & analysis for (resting-state) brain imaging. Neuroinformatics. (2016) 14:339–51. 10.1007/s12021-016-9299-4 27075850

[42] Lesh TA Tanase C Geib BR Niendam TA Yoon JH Minzenberg MJ. A multimodal analysis of antipsychotic effects on brain structure and function in first-episode schizophrenia. JAMA Psychiatry. (2015) 72:226–34. 10.1001/jamapsychiatry.2014.2178 25588194 PMC4794273

[43] Andreasen NC Pressler M Nopoulos P Miller D Ho BC. Antipsychotic dose equivalents and dose-years: a standardized method for comparing exposure to different drugs. Biol Psychiatry. (2010) 67:255–62. 10.1016/j.biopsych.2009.08.040 19897178 PMC3677042

[44] Liao J Yan H Liu Q Yan J Zhang L Jiang S. Reduced paralimbic system gray matter volume in schizophrenia: correlations with clinical variables, symptomatology and cognitive function. J Psychiatr Res. (2015) 65:80–6. 10.1016/j.jpsychires.2015.04.008 25937503

[45] Jiang Y Duan M Chen X Zhang X Gong J Dong D. Aberrant prefrontal-thalamic-cerebellar circuit in schizophrenia and depression: evidence from a possible causal connectivity. Int J Neural Syst. (2019) 29:1850032. 10.1142/S0129065718500326 30149746

[46] Zhang YY Liao JM Li QQ Zhang X Liu LJ Yan J. Altered resting-state brain activity in schizophrenia and obsessive-compulsive disorder compared with non-psychiatric controls: commonalities and distinctions across disorders. Front Psychiatry. (2021) 12:681701. 10.3389/fpsyt.2021.681701 34093290 PMC8176119

[47] Li Z Lei W Deng W Zheng Z Li M Ma X. Aberrant spontaneous neural activity and correlation with evoked-brain potentials in first-episode, treatment-naive patients with deficit and non-deficit schizophrenia. Psychiatry Res Neuroimaging. (2017) 261:9–19. 10.1016/j.pscychresns.2017.01.001 28092779

[48] Li Q Cao X Liu S Li Z Wang Y Cheng L. Dynamic alterations of amplitude of low-frequency fluctuations in patients with drug-naive first-episode early onset schizophrenia. Front Neurosci. (2020) 14:901. 10.3389/fnins.2020.00901 33122982 PMC7573348

[49] Kapur N Ellison D Smith MP McLellan DL Burrows EH. Focal retrograde amnesia following bilateral temporal lobe pathology. A neuropsychological and magnetic resonance study. Brain. (1992) 115(Pt. 1):73–85. 10.1093/brain/115.1.73 1559164

[50] Maguire EA Mummery CJ. Differential modulation of a common memory retrieval network revealed by positron emission tomography. Hippocampus. (1999) 9:54–61. 10088900 10.1002/(SICI)1098-1063(1999)9:1<54::AID-HIPO6>3.0.CO;2-O

[51] Tomadesso C Perrotin A Mutlu J Mezenge F Landeau B Egret S. Brain structural, functional, and cognitive correlates of recent versus remote autobiographical memories in amnestic Mild Cognitive Impairment. Neuroimage Clin. (2015) 8:473–82. 10.1016/j.nicl.2015.05.010 26106572 PMC4474362

[52] Marinkovic K Dhond RP Dale AM Glessner M Carr V Halgren E. Spatiotemporal dynamics of modality-specific and supramodal word processing. Neuron. (2003) 38:487–97. 10.1016/S0896-6273(03)00197-1 12741994 PMC3746792

[53] Mesulam MM Rogalski EJ Wieneke C Hurley RS Geula C Bigio EH. Primary progressive aphasia and the evolving neurology of the language network. Nat Rev Neurol. (2014) 10:554–69. 10.1038/nrneurol.2014.159 25179257 PMC4201050

[54] Chadwick MJ Anjum RS Kumaran D Schacter DL Spiers HJ Hassabis D. Semantic representations in the temporal pole predict false memories. Proc Natl Acad Sci USA. (2016) 113:10180–5. 10.1073/pnas.1610686113 27551087 PMC5018755

[55] Jimura K Konishi S Miyashita Y. Temporal pole activity during perception of sad faces, but not happy faces, correlates with neuroticism trait. Neurosci Lett. (2009) 453:45–8. 10.1016/j.neulet.2009.02.012 19429013

[56] Frith U Frith CD. Development and neurophysiology of mentalizing. Philosophical transactions of the Royal Society of London Series B, Biological sciences. (2003) 358:459–73. 10.1098/rstb.2002.1218 12689373 PMC1693139

[57] Pehrs C Zaki J Schlochtermeier LH Jacobs AM Kuchinke L Koelsch S. The temporal pole top-down modulates the ventral visual stream during social cognition. Cerebral Cortex. (2017) 27:777–92. 10.1093/cercor/bhv226 26604273

[58] McClelland JL Rogers TT. The parallel distributed processing approach to semantic cognition. Nat Rev Neurosci. (2003) 4:310–22. 10.1038/nrn1076 12671647

[59] Patterson K Nestor PJ Rogers TT. Where do you know what you know? The representation of semantic knowledge in the human brain. Nat Rev Neurosci. (2007) 8:976–87. 10.1038/nrn2277 18026167

[60] Joubert S Gour N Guedj E Didic M Gueriot C Koric L. Early-onset and late-onset Alzheimer's disease are associated with distinct patterns of memory impairment. Cortex. (2016) 74:217–32. 10.1016/j.cortex.2015.10.014 26694580

[61] Campo P Poch C Toledano R Igoa JM Belinchon M Garcia-Morales I. Visual object naming in patients with small lesions centered at the left temporopolar region. Brain Struct Funct. (2016) 221:473–85. 10.1007/s00429-014-0919-1 25342238

[62] Petrides M. Lateral prefrontal cortex: architectonic and functional organization. Philos Trans R Soc Lond Ser B Biol Sci. (2005) 360:781–95. 10.1098/rstb.2005.1631 15937012 PMC1569489

[63] Muller NG Knight RT. The functional neuroanatomy of working memory: contributions of human brain lesion studies. Neuroscience. (2006) 139:51–8. 10.1016/j.neuroscience.2005.09.018 16352402

[64] Koechlin E Summerfield C. An information theoretical approach to prefrontal executive function. Trends Cogn Sci. (2007) 11:229–35. 10.1016/j.tics.2007.04.005 17475536

[65] Bortolato B Miskowiak KW Kohler CA Vieta E Carvalho AF. Cognitive dysfunction in bipolar disorder and schizophrenia: a systematic review of meta-analyses. Neuropsychiatr Dis Treat. (2015) 11:3111–25. 10.2147/NDT.S76700 26719696 PMC4689290

[66] Poppe AB Barch DM Carter CS Gold JM Ragland JD Silverstein SM. Reduced frontoparietal activity in schizophrenia is linked to a specific deficit in goal maintenance: a multisite functional imaging study. Schizophr Bull. (2016) 42:1149–57. 10.1093/schbul/sbw036 27060129 PMC4988742

[67] Rizzolatti G Craighero L. The mirror-neuron system. Annu Rev Neurosci. (2004) 27:169–92. 10.1146/annurev.neuro.27.070203.144230 15217330

[68] Jani M Kasparek T. Emotion recognition and theory of mind in schizophrenia: a meta-analysis of neuroimaging studies. World J Biol Psychiatry. (2018) 19:S86–96. 10.1080/15622975.2017.1324176 28449613

[69] Cook R Bird G Catmur C Press C Heyes C. Mirror neurons: from origin to function. Behav Brain Sci. (2014) 37:177–92. 10.1017/S0140525X13000903 24775147

[70] Sun F Zhao Z Lan M Xu Y Huang M Xu D. Abnormal dynamic functional network connectivity of the mirror neuron system network and the mentalizing network in patients with adolescent-onset, first-episode, drug-naive schizophrenia. Neurosci Res. (2021) 162:63–70. 10.1016/j.neures.2020.01.003 31931027

[71] Choe E Lee TY Kim M Hur JW Yoon YB Cho KK. Aberrant within- and between-network connectivity of the mirror neuron system network and the mentalizing network in first episode psychosis. Schizophr Res. (2018) 199:243–9. 10.1016/j.schres.2018.03.024 29599093

[72] Xu J Lyu H Li T Xu Z Fu X Jia F. Delineating functional segregations of the human middle temporal gyrus with resting-state functional connectivity and coactivation patterns. Hum Brain Mapp. (2019) 40:5159–71. 10.1002/hbm.24763 31423713 PMC6865466

[73] Schurz M Radua J Aichhorn M Richlan F Perner J. Fractionating theory of mind: a meta-analysis of functional brain imaging studies. Neurosci Biobehav Rev. (2014) 42:9–34. 10.1016/j.neubiorev.2014.01.009 24486722

[74] Schurz M Tholen MG Perner J Mars RB Sallet J. Specifying the brain anatomy underlying temporo-parietal junction activations for theory of mind: a review using probabilistic atlases from different imaging modalities. Hum Brain Mapp. (2017) 38:4788–805. 10.1002/hbm.23675 28608647 PMC6867045

[75] Lee JS Kim CY Joo YH Newell D Bouix S Shenton ME. Increased diffusivity in gray matter in recent onset schizophrenia is associated with clinical symptoms and social cognition. Schizophr Res. (2016) 176:144–50. 10.1016/j.schres.2016.08.011 27554199 PMC5392041

