# Supplementary material for: Associations of Neurocognition and Social Cognition With Brain Structure and Function in Early-Onset Schizophrenia
Source: Front Psychiatry. 2022 Feb 10;13:798105. doi: 10.3389/fpsyt.2022.798105 (PMC8866448; doi:10.3389/fpsyt.2022.798105)
Supplement: Supplementary file 1 [file Data_Sheet_1.docx]

Supplementary Material

1. **Supplementary Table 1.** Behavioral outcomes of the cognitive tests

| Characteristics | Mean ± SD | Range |
| --- | --- | --- |
| AVLT performance | | |
| Immediate recall | 7.45 ±2.43 | 2.80-14 |
| Short-term delayed recall | 8.09 ±2.98 | 2-14 |
| Long-term delayed recall | 7.05 ±3.14 | 0-13 |
| Long-term delayed recognition | 10.77 ±2.70 | 5-15 |
| VFT | 35.26 ±8.53 | 12-60 |
| Digit span task performance | | |
| Digit span forward | 9.58 ±1.48 | 5-13 |
| Digit span backward | 4.72 ±1.59 | 2-9 |
| Stroop Color Word Test performance | | |
| Stroop-dot | 16.22 ±4.25 | 10.2-28 |
| Stroop-word | 19.59 ±7.25 | 10.9-49 |
| Stroop-color word | 29.55 ±9.67 | 13-60 |
| TOM-PST performance | | |
| Primary beliefs | 1.67 ±0.71 | 0-2 |
| Primary false beliefs | 2.35 ±0.78 | 1-3 |
| Secondary beliefs | 1.98 ±0.15 | 1-2 |
| Secondary false beliefs | 2.12 ±0.96 | 0-3 |
| Tertiary false beliefs | 2.12 ±0.91 | 0-3 |
| Sense of reality | 1.88 ±0.39 | 0-2 |
| Understanding reciprocity | 2.65 ±0.57 | 1-3 |
| Understanding deception | 2.56 ±0.73 | 0-3 |
| Detecting deception | 1.77 ±0.53 | 0-2 |
| Total score of TOM | 48.51 ±8.68 | 24-59 |
| AIHQ performance | | |
| IHB | 1.95 ±0.82 | 1-4 |
| IBB | 2.91 ±1.00 | 1-5.33 |
| IAB | 2.05 ±0.73 | 1-4.40 |
| AmHB | 1.77 ±0.70 | 1-3.40 |
| AmBB | 2.48 ±0.93 | 1-5.33 |
| AmAB | 1.79 ±0.53 | 1-3.20 |
| AcHB | 1.35 ±0.65 | 1-4 |
| AcBB | 1.90 ±0.86 | 1-5.33 |
| AcAB | 1.65 ±0.75 | 1-4.60 |
| THB | 5.07 ±1.73 | 3-10.80 |
| TBB | 7.30 ±2.48 | 3-16 |
| TAB | 5.48 ±1.63 | 3-11 |

SD, standard deviation; AVLT, auditory verbal learning test; VFT, verbal fluency test; TOM-PST, theory of mind picture-sequencing task; AIHQ, ambiguous intentions hostility questionnaire; IHB, intentional hostility bias; IBB, intentional blame bias; IAB, intentional aggression bias; AmHB, ambiguous hostility bias; AmBB, ambiguous blame bias; AmAB, ambiguous aggression bias; AcHB, accidental hostility bias; AcBB, accidental blame bias; AcAB, accidental aggression bias; THB, total scores of hostility bias; TBB, total scores of blame bias; TAB, total scores of aggression bias.

1. **Supplementary Table 2.** Correlation between neurocognition and GMV before adjusting for the outliers of outcomes

| Neurocognition | Brain region | *pr* | *P* |
| --- | --- | --- | --- |
| AVLT-immediate recall | Left temporal pole | 0.626 | <0.001 |
| VFT | Left temporal pole: middle temporal gyrus | 0.635 | <0.001 |
| Stroop-word | Right middle frontal gyrus | 0.629 | <0.001 |

Abbreviations: GMV, gray matter volume; AVLT, auditory verbal learning test; VFT, verbal fluency test; *pr*, partial correlation coefficient.

1. **Supplementary Table 3.** Correlation between social cognition and ALFF before adjusting for the outliers of outcomes

| Social cognition | Brain region | *pr* | *P* |
| --- | --- | --- | --- |
| TOM-sense of reality | Left hippocampus | -0.647 | <0.001 |
| TOM-sense of reality | Left precentral gyrus | -0.724 | <0.001 |
| AIHQ- AcHB | Left fusiform gyrus | 0.611 | <0.001 |
| AIHQ- AcHB | Right middle temporal gyrus | 0.624 | <0.001 |
| AIHQ- AcAB | Left fusiform gyrus | 0.656 | <0.001 |
| AIHQ- AcAB | Left precentral gyrus | 0.633 | <0.001 |
| TAB | Left fusiform gyrus | 0.603 | <0.001 |

Abbreviations: ALFF, amplitude of low-frequency fluctuation; TOM, theory of mind; AIHQ, ambiguous intentions hostility questionnaire; AcHB, accidental hostility bias; AcAB, accidental aggression bias; TAB, total scores of aggression bias; *pr*, partial correlation coefficient.

1. **Supplementary Table 4.** Correlation between neurocognition and GMV after adjusting for the antipsychotics effect

| Neurocognition | Brain region | *pr* | *P* |
| --- | --- | --- | --- |
| AVLT-immediate recall | Left temporal pole | 0.639 | <0.001 |
| VFT | Left temporal pole: middle temporal gyrus | 0.683 | <0.001 |
| Stroop-word | Right middle frontal gyrus | 0.453 | 0.005 |

Abbreviations: GMV, gray matter volume; AVLT, auditory verbal learning test; VFT, verbal fluency test; *pr*, partial correlation coefficient.

1. **Supplementary Table 5.** Correlation between social cognition and ALFF after adjusting for the antipsychotics effect

| Social cognition | Brain region | *pr* | *P* |
| --- | --- | --- | --- |
| TOM-sense of reality | Left precentral gyrus | -0.430 | 0.008 |
| AIHQ- AcHB | Right middle temporal gyrus | 0.397 | 0.018 |
| AIHQ- AcAB | Left precentral gyrus | 0.303 | 0.072 |

Abbreviations: ALFF, amplitude of low-frequency fluctuation; TOM, theory of mind; AIHQ, ambiguous intentions hostility questionnaire; AcHB, accidental hostility bias; AcAB, accidental aggression bias; *pr*, partial correlation coefficient.

1. **Supplementary Table 6.** Correlation between neurocognition and GMV after adjusting for the age of onset of schizophrenia

| Neurocognition | Brain region | *pr* | *P* |
| --- | --- | --- | --- |
| AVLT-immediate recall | Left temporal pole | 0.617 | <0.001 |
| VFT | Left temporal pole: middle temporal gyrus | 0.654 | <0.001 |
| Stroop-word | Right middle frontal gyrus | 0.475 | 0.003 |

Abbreviations: GMV, gray matter volume; AVLT, auditory verbal learning test; VFT, verbal fluency test; *pr*, partial correlation coefficient.

1. **Supplementary Table 7.** Correlation between social cognition and ALFF after adjusting for the age of onset of schizophrenia

| Social cognition | Brain region | *pr* | *P* |
| --- | --- | --- | --- |
| TOM-sense of reality | Left precentral gyrus | -0.336 | 0.042 |
| AIHQ- AcHB | Right middle temporal gyrus | 0.376 | 0.026 |
| AIHQ- AcAB | Left precentral gyrus | 0.370 | 0.026 |

Abbreviations: ALFF, amplitude of low-frequency fluctuation; TOM, theory of mind; AIHQ, ambiguous intentions hostility questionnaire; AcHB, accidental hostility bias; AcAB, accidental aggression bias; *pr*, partial correlation coefficient.
